# The Tdap Vaccination in Pregnancy: Results of a Healthy Equity Audit on Coverage Trends and Their Determinants in the Reggio Emilia Province (Italy)

**DOI:** 10.3390/vaccines13030251

**Published:** 2025-02-27

**Authors:** Laura Bonvicini, Filomena Giulia Sileo, Benedetta Riboldi, Eufemia Bisaccia, Marco Tamelli, Daniela Bertani, Silvia Cilloni, Luca Ghirotto, Paolo Giorgi Rossi

**Affiliations:** 1 Epidemiology Unit, Azienda Unità Sanitaria Locale-IRCCS di Reggio Emilia, 42122 Reggio Emilia, Italy; laura.bonvicini@ausl.re.it (L.B.); paolo.giorgirossi@ausl.re.it (P.G.R.); 2 Prenatal Medicine Unit, Obstetrics and Gynecology Unit, Department of Medical and Surgical Sciences for Mother, Child and Adult, University of Modena and Reggio Emilia, 41121 Modena, Italy; 3 Azienda Unità Sanitaria Locale-IRCCS di Reggio Emilia, 42122 Reggio Emilia, Italy; benedetta.riboldi@ausl.re.it; 4 Public Health Unit, Azienda Unità Sanitaria Locale-IRCCS di Reggio Emilia, 42122 Reggio Emilia, Italy; eufemia.bisaccia@ausl.re.it (E.B.); marco.tamelli@ausl.re.it (M.T.); silvia.cilloni@ausl.re.it (S.C.); 5 Salute Donna, Azienda Unità Sanitaria Locale-IRCCS di Reggio Emilia, 42122 Reggio Emilia, Italy; daniela.bertani@ausl.re.it; 6 Qualitative Research Unit, Azienda Unità Sanitaria Locale-IRCCS di Reggio Emilia, 42122 Reggio Emilia, Italy; luca.ghirotto@ausl.re.it

**Keywords:** vaccine, Tdap, immunization, pregnancy, maternal vaccination

## Abstract

**Background/Objectives:** The Italian National Plan for Vaccine Prevention 2017–2019 recommended tetanus, diphtheria, and acellular pertussis vaccines (Tdap) for pregnant women, irrespectively of their immunization history. This study aims to describe the coverage rate trends for Tdap vaccination in pregnancy and evaluate the differences by socioeconomic status. **Methods**: This is a retrospective analysis within a health equity audit of the Local Health Authority of Reggio Emilia on vaccination in pregnancy from 2018 (a local vaccination campaign) to 2023. All women residents in our area who gave birth during that period were included and linked to the electronic Registry of Immunization Service. The vaccination coverage in pregnant women was analyzed over time and stratified by pregnant women’s sociodemographic and obstetric characteristics. **Results**: The coverage of Tdap in pregnant women of the Province of Reggio Emilia increased from 15.9% in 2018 to 53.9% in 2023. The coverage was higher among Italians, women with higher educational levels (aPR 1.49 (CI95%1.41–1.57)), within 31–35 years of age (aPR 1.37 (CI95% 1.28–1.46)), occupied, nulliparous (aPR multiparous vs nulliparous: 0.76 (0.74; 0.78)), and followed in the private sector (aPR 1.07 (1.03–1.11)). Inequalities in coverage increased during the study period for women assisted in the private sector, while decreased or remained stable for women assisted in the context of public services. **Conclusions**: The vaccination promotion campaign in Reggio Emilia helped increase Tdap coverage in pregnancy from 16 to 53%. Nevertheless, the coverage rates of the most disadvantaged women are still several points lower than the average.

## 1. Introduction

Maternal immunization represents a public health strategy to protect both mother and fetus/newborn against vaccine-targeted infections [1]. Maternal immunoglobulins G (IgG) can cross the placenta from 17 weeks of gestation onward, and their concentration increases with advancing gestation, reaching its zenith at around 40 weeks [1]. Therefore, administering vaccines to pregnant women during the second or third trimester of pregnancy has become a relatively new strategy to provide fetal and neonatal protection through the trans-placental transfer of maternal antibodies [2]. Over the last decades, maternal administration of the tetanus vaccine has effectively reduced neonatal tetanus [3], and the WHO launched the Maternal and Neonatal Tetanus Elimination (MNTE) initiative [4].

Similarly, to reduce the burden of pertussis in infants, the Centers for Disease Control and Prevention (CDC) in the United States recommended that pregnant women should receive tetanus, diphtheria, and acellular pertussis (Tdap) vaccines in every pregnancy between 27 and 36 weeks of gestation [5]; this recommendation was also adopted by other health organizations [6,7].

Despite the scientific evidence on vaccine efficacy and safety [8,9], the number of under-vaccinated or unvaccinated subjects in Italy has alarmingly increased over the last few years [10]. Therefore, a new National Plan for Vaccine Prevention (NPVP) 2017–2019 was approved in Italy [11] to counteract this trend. The NPVP recommends, among other recommendations and obligations, the Tdap vaccination for pregnant women at 28 weeks of gestation during each pregnancy, irrespective of the number of previous doses received [12]. The NPVP recommends strict monitoring of vaccine coverage and investigating the possible barriers and the existing inequalities.

In 2018, the Local Health Authority of Reggio Emilia (LHA) implemented a campaign to increase anti-pertussis coverage in pregnancy, with information leaflets, flyers, and gadgets for women, awareness raising for providers, and a communication campaign in local media. In 2024 within the activities of the Regional Preventive Plan, the LHA started a Health Equity Audit (HEA) on vaccination in pregnancy. A group of professionals with diverse backgrounds is engaged to analyze the context, highlight inequalities, identify priorities and appropriate interventions, and monitor and document the process.

The reported analyses aim to describe the coverage rate trends for Tdap vaccination and evaluate the differences by socioeconomic status among pregnant women from 2018 to 2023. Furthermore, we investigate whether the setting where women receive assistance and the healthcare operator who provides the care during pregnancies influence the overall coverage and the differences across socioeconomic strata.

## 2. Materials and Methods

### 2.1. Setting and Design

The Reggio Emilia Province, in northern Italy, has 532,000 inhabitants and about 3500 pregnancies per year (with a slight decline over the study period). The Local Health Authority (LHA), the local public entity of the Italian National Health Service, provides hospital, outpatient, primary, and preventive care to the entire population residing in the province [13].

Pertussis cases in the province of Reggio Emilia, in the total population and in young children in the first year of life, from 2012 to 2023 are reported in Table 1. The data were collected by the local infectious disease control services. There were no cases of diphtheria reported in the period, and one case of tetanus in one woman was reported.

Recommended vaccines are provided for free to all the people residing in Italy independently from citizenship and insurance coverage.

The Emilia-Romagna Regional Prevention Plan 2020–2025 [14] requires that each LHA conducts HEA on selected preventive interventions at risk of inequality. The Reggio Emilia LHA, based on insights from COVID-19 immunization campaigns, included vaccinations in adulthood in the preventive interventions to be the object of the HEA and started an Audit on Tdap vaccination in pregnancy.

During the vaccination campaign in 2018 to promote Tdap vaccination adhesion, a multi-sectorial program was set up that included the actions described in Box 1.

Box 1 **Activities implemented for the information and vaccination promotion campaign.**

- The definition and approval of a Standard Operating Procedure (SOP) regarding all the vaccinations available in pregnancy, including Tdap, with all the information (timing, place for the administration, etc.) for the specialist workers.
- An informative vaccination booklet for all pregnant women was also distributed to the obstetricians, midwives, and General Practitioners (GP) working in the healthcare centers of the province.
- A press release in local newspapers to promote the campaign.
- The distribution of a small gadget, a pin, or a keyholder with the claim “I got vaccinated” to all women receiving the vaccine.
- The promotion of vaccination on the LHA internet page and on social media with the hashtag #miproteggoperproteggerci

The CedAP database is a national registry containing all the epidemiological, sociodemographic, and health data regarding birth events, including data about congenital malformations and perinatal deaths, established by the Italian Ministry of Health in 2001 (Decree 16 July 2001, n° 349). Its use was started in the Emilia Romagna region in 2002, with additional information to implement the regional database; the data from every province in Emilia Romagna are acquired and analyzed every six months [15].

To compute the Tdap vaccine coverage in pregnancy, the list of women residing in the Province of Reggio Emilia from 2018 to 2023 from the CedAP registry was linked to the electronic Registry of Immunization using an anonymous unique personal identifier.

All the vaccinations, including the Tdap vaccine in pregnant women, are recorded in the electronic Registry of Immunization Service of the Public Health Department of the Local Health Unit of Reggio Emilia. This database includes the recipient’s data for each administered vaccine dose, including age, residence, and place where the vaccination was carried out.

The vaccination status was analyzed over time and stratified by the sociodemographic and obstetric characteristics of pregnant women and their partners. Age, citizenship, marital status, educational level, occupational status, previous conceptions, number of previous deliveries, and number of prior miscarriages and/or termination of pregnancies were extracted from CedAP. Additional obstetric variables were extracted from the CedAP and included in the analysis, such as the type of assistance received during pregnancy (i.e., private practice vs. public care), the health workers in charge of the pregnancy (i.e., midwife alone, obstetrician and midwife, obstetrician alone), the number of checks, and the gestational age at the first antenatal check and the type of pregnancy (low-risk vs. high-risk pregnancy) [16].

### 2.2. Statistical Analysis

The study included only women with pregnancies lasting at least 27 gestational weeks (the gestational age inferior limit from when the vaccination should be administered) and resident in the Province of Reggio Emilia.

Descriptive analyses were performed, and multivariate models calculated the adjusted prevalence rate ratios (aPR) and the related 95% confidence intervals. The analyses were stratified by the type of service and health worker who followed the pregnancy and by parity.

### 2.3. Ethics

The reported analyses are part of a Health Equity Audit of the Local Health Authority of the Reggio Emilia Province, including the Regional Preventive Plan (PRP) 2020–2025. The Regional Coordination of the Prevention Plan has approved the Audit. Women sign a data treatment information for vaccination. Analyses were conducted on pseudo-anonymized data at the local level within the duties of the LHA.

## 3. Results

During the study period, the number of Tdap vaccinations among 14–50-year-old women ranged from 4368 in 2021 (4.0%) to 543 (0.5%) in 2020, with a mean coverage of 2.4%. This drop in the overall volume of Tdap vaccinations provided by the health services was expected in the year of the COVID-19 pandemic. On the contrary, the positive trend observed for the coverage of pregnant women did not show any change during the pandemic. In the same years, the total number of deliveries occurring at ≥27 weeks of gestation was 21,115; the number of childbirths, however, decreased from 3750 in 2018 to 3297 in 2023. When looking at the coverage of Tdap in pregnant women in the Province of Reggio Emilia, this increased from 15.9% to 53.9% during the study period, reaching its highest value (54.8%, *n* = 1837/3350) in 2022, as shown in Table 2.

### 3.1. Coverage and Sociodemographic Variables

**Figure 1:**
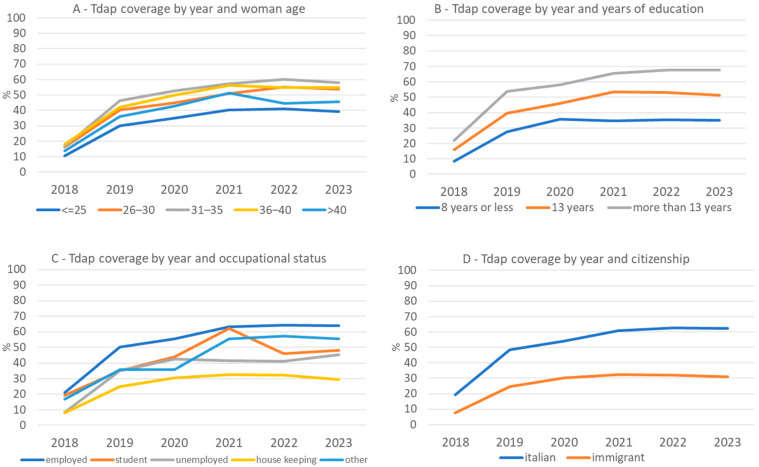
Tdap coverage according to year and maternal age (**A**), education level (**B**), occupational status (**C**), and citizenship (**D**).

The coverage time trends in the study period by the main sociodemographic variables are presented in Figure 1. Coverage increased over the years in all age groups, with the highest uptake in women between 31 and 35 years of age and the lowest in the two extreme age groups, i.e., among women ≤25 or >40 years of age, as shown in Figure 1A. The coverage was also higher among women with higher education levels (Figure 1B).

When looking at occupational status (Figure 1C), the highest coverage (up to 64.4% in 2022) was among employed women, while the lowest coverage was among housekeeping women. Finally, although coverage increased over time in both groups (Figure 1D), the gap in terms of coverage between Italian women and women of other citizenships increased over time.

The majority of pregnancies (69.4%, n = 14,641) were classified as physiological. Sixty-six percent of pathological pregnancies were followed in the public sector. Eighty-nine percent of midwifery-led physiological pregnancies were followed in the public sector (only 11% in private settings). Physiological pregnancies followed by an obstetrician amounted to 28% in the public sector and 72% in the private sector.

### 3.2. Coverage and Obstetric Variables

**Figure 2:**
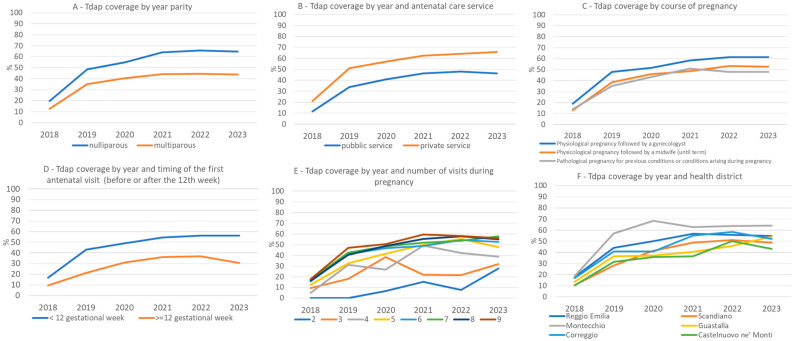
Tdap coverage according to year and parity (**A**), antenatal care service (**B**), course of pregnancy (**C**), timing of the first antenatal check (**D**), number of checks in pregnancy (**E**), and health district (**F**).

Figure 2 shows the coverage of Tdap according to obstetric variables.

More in detail, nulliparous women had a higher uptake of Tdap than multiparous, with an increasing gap between the two groups over time (Figure 2A). However, the Tdap coverage increased over the years in both private and public services, with the percentage of women receiving the vaccination being higher among women in the private sector in the whole period (Figure 2B). Women with a physiological course of the pregnancy followed up by an obstetrician had a higher uptake of Tdap than women with a physiological course of the pregnancy but followed up by a midwife. The lowest uptake was in the group of women with a pathological course of pregnancy (Figure 2C).

The coverage of Tdap vaccination was higher in women receiving the first antenatal check before 12 weeks (Figure 2D) and receiving at least five antenatal checks during the pregnancy (Figure 2E).

There were differences in the vaccination coverage by health district, with the Montecchio district having the highest (Figure 2F).

### 3.3. Multivariable Models

The data regarding the uptake of Tdap according to sociodemographic and obstetric variables and their interactions are presented in Table 3.

When correcting the model for parity and age, the women with a physiological course of the pregnancy and followed by an obstetrician were more likely to be vaccinated (aPR 1.10, 95%CI 1.06–1.14) compared to women with a pathological course of the pregnancy.

Furthermore, nulliparity and a maternal age between 26 and 35 years were also associated with higher odds of vaccination.

When adjusting for citizenship (Italian vs. others), women with a physiological pregnancy course and who were followed by an obstetrician showed the same odds of vaccination (aPR 1.01, 95%CI 0.97–1.04). The model adjusted also for educational level showed that the propensity to be vaccinated increased with an increasing educational level. The interaction between educational level and citizenship was significant and showed a minor effect on educational level among women with citizenships other than Italian.

Considering the sociodemographic and obstetric characteristics and antenatal care indicators (Table 4), a higher vaccination rate was observed in the private compared to the public sector (aPR private vs public: 1.07 95%CI 1.03–1.11). A higher vaccination rate was observed in the Montecchio district (Montecchio vs Reggio Emilia 1.20 95%CI 1.15–1.56). The high vaccination coverage in the Montecchio district was appreciable only in the public (aPR Montecchio vs Reggio Emilia 1.43 (1.36–1.51) and not in the private sector (aPR 0.87–0.99). In the public sector in Montecchio, women with a medium or low educational level (≤13 years) had higher coverage (55.6% and 59.8%, respectively) than in the other districts (59.8% and 55.7%, respectively); similarly, in the Montecchio district, coverage in non-Italian women was higher (46.5%) than in other districts (23.1%).

The analysis of two indicators of antenatal care (i.e., the number of antenatal checks—in physiological pregnancies—and the gestational age at the first check) showed that the propensity to be vaccinated increased in women compliant with the standard of care (considering all sociodemographic and obstetric variables) (aPR eight or more visits vs. less than four: 1.69 95%CI 1.27; 2.24—aPR first visit after 12 weeks of gestation vs first visit before 12 weeks: 0.76 95%CI 0.69; 0.84).

**Figure 3:**
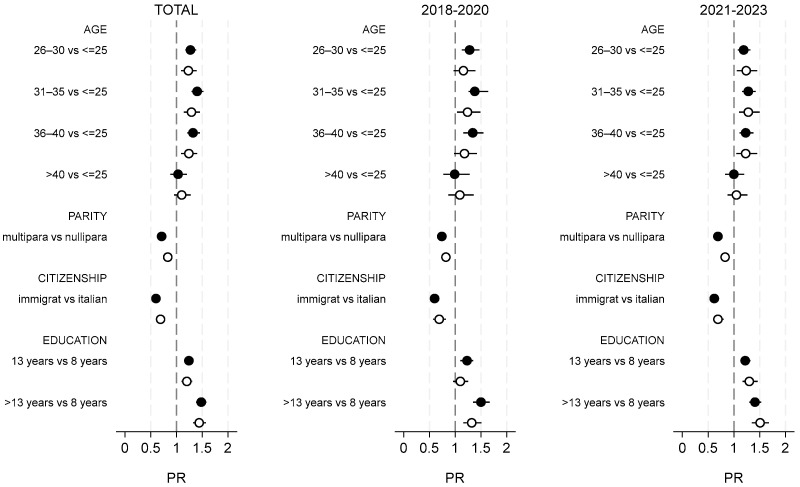
PR of Tdap uptake according to several variables stratified by type of setting and period (public vs. private sector).

Figure 3 shows that by stratifying by care setting (private vs. public sector) the relative risk of Tdap uptake differed only slightly according to the different sociodemographic and obstetric variables. Comparing the two periods, an increase in differences by educational level was observed in the private sector and not in the public sector, where differences decreased slightly.

## 4. Discussion

### 4.1. Key Findings

This study shows that the campaign, including information leaflets, flyers, gadgets for women, awareness raising for providers, and the communication campaign in local media, was influential in raising the Tdap coverage in pregnancy, going from 16% to 54% in 5 years. Surprisingly, the pandemic did not affect the positive trend in Tdap vaccine coverage for pregnant women, despite a large decrease in the overall Tdap doses administered in adults. However, the rates of Tdap coverage in the last year of the study period (2023) were lower than in 2022. Among the variables that possibly influenced the Tdap uptake, nulliparity, a maternal age of 31–35 years, Italian citizenship, and a higher education level were associated with higher Tdap coverage. The setting of care had an impact on coverage and on inequalities.

### 4.2. Interpretation of the Study Findings and Comparison with the Published Literature

The National Immunization Prevention Plan promoted by the Ministry of Health in 2017–2019 recommended both influenza and Tdap vaccines during pregnancy in Italy. However, despite the recommendations, maternal vaccine uptake for pertussis was reported to be only 5% among pregnant women involved in a multicenter Italian survey [17].

The role of a promotion campaign to improve vaccination uptake was recently evaluated by Cremer and colleagues [18], with their interventional study showing no differences after the campaign; however, the information campaign was not aimed at the general population as in our study, but only at the obstetricians. The positive effect of a campaign targeting the public was shown in the Stockport (UK) experience [19], where, after a local awareness campaign about the influenza vaccine, the uptake rates increased in both low- and high-risk pregnant women.

In our area, the vaccination promotion campaign started in 2018 and resulted in an almost threefold increase in the absolute number of vaccinations among pregnant women in the following year (2019). The coverage rates continued to grow in the following two years (2020 and 2021), but they reached a plateau at around 53–54% after that, with no significant increase in the last two years.

Difficult to say whether this plateau represents the saturation of women who have a high propensity to be vaccinated.

Vilca et al. [17] showed that the main barrier to vaccination was any healthcare provider’s lack of immunization advice. At the same time, the willingness to protect their offspring was the primary facilitator. Furthermore, several studies have already shown the critical role of healthcare professionals (midwives and obstetricians) in the decision-making process of pregnant women [20,21]. In our cohort, the low-risk pregnant women followed by midwives were less likely to get vaccinated than those followed by an obstetrician. This is consistent with several studies reporting that midwives are less likely to discuss and recommend vaccinations than other healthcare providers [22,23,24], mainly because of safety concerns [25].

Our study confirms previous findings [17,22] that a low-to-middle education level is significantly associated with lower uptakes of the Tdap vaccine and that women with diverse cultural and linguistic backgrounds might have additional barriers. Furthermore, our data show that the gap in the uptake of vaccination between Italian women and women holding other citizenships was not reduced despite the increase in coverage in both groups, suggesting that the interventions put in place during the campaign were not addressing the specific barriers underlying these differences. Our data are consistent with the data provided by Bonito et al. [26] in another Italian region and by Krishnaswamy et al. [27] in Australia. It is worth noting that, in infant and adolescent vaccinations, in Italy, children with highly educated mothers do not have advantages in coverage [28].

### 4.3. Strengths and Limitations of the Study

The main strength of our study is that all women delivering at ≥27 weeks during the study period could be included in the study and linked to the electronic Registry of Immunization Service of the Public Health Department of Reggio Emilia Province. Therefore, sociodemographic and obstetric variables were available for the whole cohort of women eligible for vaccination. Another strength is the large number of deliveries included over a wide range of time.

However, several limitations should be acknowledged. First, the study’s retrospective nature with no information on women’s attitudes toward vaccination and no information regarding the reasons for declining vaccination. Secondly, temporal trends other than the vaccination campaign might have had an impact and could not be evaluated in this study. Nevertheless, despite the effect of the COVID-19 pandemic in 2020 being appreciable, the observed time trends are so large that they can hardly be due to social environmental confounders. Another limitation is that the antibody titer variation in the pregnant women and newborns were not available, as this is not routinely assessed and, therefore, we could not assess the real effectiveness of the campaign in increasing the protection. Furthermore, the impact of the vaccine on the burden of the disease is difficult to assess in a local implementation due to the low incidence of the disease. Finally, not all the campaign activities were documented quantitatively; therefore, the total effort, in terms of economic and human resources, cannot be assessed

### 4.4. Implications for Clinical Practice and Research

The campaign reached a plateau at about 50%, with some disadvantaged groups still having coverage below 30%. Strategies to propose immunization during pregnancy should be designed to be sustainable and not one-time occasions. Most of the interventions put in place in the reported campaign are durable. Nevertheless, those targeting directly the public, such as press releases and local media messages, need to be repeated often and are resource consuming. Furthermore, interventions should be rethought to act on the specific barriers hampering the participation of immigrant women, who are about one-third of the pregnant women and are a strongly disadvantaged group. Pregnant women have many contacts with HCPs in the time window suitable for vaccination. Therefore, it is logical to invest in these opportunities to promote vaccination as well as other preventive interventions. However, too many health promotion messages targeting vulnerable pregnant women may be confusing and distract the woman from the main scope of the pregnancy assistance. Despite the previous negative experiences confirmed in this study, it is vital to study interventions that can effectively use this channel, avoiding information overload for the woman.

To overcome these problems and promote vaccination among pregnant women, this HEA conducted in the Reggio Emilia province continues to investigate stakeholders’ perceptions of vaccination in pregnancy through qualitative investigations.

Furthermore, within this HEA, two specific training events for obstetricians and midwives are planned to improve HCPs’ knowledge about vaccination in pregnancy and how to promote it among pregnant women. These training sessions will be held by medical staff and a psychologist who will inform HCPs about the vaccine desirable and undesirable effects and train them on motivational counselling applied to vaccination [29].

In our region, tetanus and diphtheria incidence is very low in mothers and newborns; therefore, we cannot expect any impact on their burden due to the increase in vaccination coverage. On the other hand, pertussis in newborns and toddlers is still a public health problem. Data from the infectious disease information system show a decreasing trend during the study period. In 2024, a peak in pertussis cases [30] was observed nationally and internationally. In Reggio Emilia, preliminary data for 2024 recorded 157 cases, 14 of which in children under one year of age. In the area of Reggio Emilia, pertussis occurred in six children between 0 and 3 months of age and in none of them the mother was vaccinated during pregnancy as recommended. The hospitalization rate among vaccinated people was 4%, while, among unvaccinated people, it was 60%. The occurrence of large, international outbreaks highlights the need of vaccination and its importance to reduce the burden of disease.

## 5. Conclusions

The campaign in Reggio Emilia to increase vaccination uptake during pregnancy increased coverage from 16 to 53%. Nevertheless, a plateau has been reached, and the coverage rates of the most disadvantaged women are still several points lower than the average. Future interventions should aim to increase coverage among all pregnant women since the reached coverage is still low. At the same time, the gap between Italians and immigrants and between women with high and low educational levels is unacceptable for a public and universalistic health service, and targeted interventions should be designed, considering the scarce awareness observed among HCPs about the beneficial effects of maternal immunization for both mother and newborn.

## Figures and Tables

**Table 1:** Pertussis cases in the total population and young children in the province of Reggio Emilia from 2012 to 2023.

**Year**		2012	2013	2014	2015	2016	2017	2018	2019	2020	2021	2022	2023	TOTAL
**Total Pertussis Cases**	**N**	14	5	17	14	67	55	15	23	8	0	2	1	378
**Cases in Children Between 0 and 1 Years Old**	**N**	0	2	1	2	3	17	2	4	0	0	1	0	46
	**%**	0.0	40.0	5.9	14.3	4.5	30.9	13.3	17.4	0.0	0.0	50.0	0.0	12.2

**Table 2:** Coverage rates of the Tdap vaccine in the general and pregnant population of Reggio Emilia from 2018 to 2023.

Year	Women 14–50 Years Old (N) *	Tdap Vaccination °		Childbirths (N) **	Tdap Vaccination	
		N	%		N	%
2018	115,220	2331	2.0	3750	595	15.9
2019	114,254	3249	2.8	3699	1528	41.3
2020	113,189	543	0.5	3522	1669	47.4
2021	111,528	3559	3.2	3497	1861	53.2
2022	110,000	2200	2.0	3350	1837	54.8
2023	109,789	4368	4.0	3297	1778	53.9

* Source: ISTAT without women childbirths from pregnancies at least 27 weeks long; ° without Tdap vaccination administered during pregnancy; ** source: CedAP, childbirths from pregnancies at least 27 weeks long.

**Table 3:** Multivariable models of Tdap vaccine uptake in the pregnant population of Reggio Emilia from 2018 to 2023.

			Model 1: Adjusted by Course of Pregnancy	Model 2: Adjusted by Course of Pregnancy, Age, Parity	Model 3: Adjusted by Course of Pregnancy, Age, Parity, Citizenship	Model 4: Adjusted by Course of Pregnancy, Age, Parity, Citizenship, Educational Level
	N (%)		PR (95%CI)	PR (95%CI)	PR (95%CI)	PR (95%CI)
Course of pregnancy						
Physicological pregnancy followed by a midwife (until term)	6248	29.6	1.00	1.00	1.00	1.00
Physiological pregnancy followed by an obstetrician	8395	39.8	1.18 (1.13; 1.22)	1.10 (1.06; 1.14)	1.01 (0.97; 1.04)	1.00 (0.96; 1.03)
Pathological pregnancy for previous conditions or conditions arising during pregnancy	6472	30.7	0.97 (0.93; 1.01)	0.95 (0.91; 0.99)	0.95 (0.91; 0.98)	0.97 (0.93; 1.07)
**Age**						
≤25	2331	11.0		1.00	1.00	1.00
26–30	5754	27.3		1.45 (1.36; 1.55)	1.36 (1.27; 1.45)	1.26 (1.18; 1.35)
31–35	7283	34.5		1.72 (1.61; 1.84)	1.54 (1.44; 1.64)	1.37 (1.28; 1.46)
36–40	4649	22.0		1.71 (1.59; 1.83)	1.49 (1.39; 1.59)	1.31 (1.22; 1.40)
>40	1098	5.2		1.42 (1.29; 1.56)	1.24 (1.12; 1.36)	1.11 (1.02; 1.22)
**Parity**						
Nullipara	10,006	47.4		1.00	1.00	1.00
Multipara	11,109	52.6		0.66 (0.64; 0.69)	0.73 (0.71; 0.75)	0.76 (0.74; 0.78)
**Citizenship**						
Italians	15,209	72.0			1.00	1.00
Others	5906	28.0			0.56 (0.53; 0.59)	0.60 (0.57; 0.62)
**Educational level (years)**						
≤8	4675	22.1				1.00
13	9500	45.0				1.24 (1.18; 1.30)
>13	6940	32.9				1.49 (1.41; 1.57)

PR: prevalence rate ratio.

**Table 4:** Multivariable models of Tdap vaccine uptake in the pregnant population of Reggio Emilia considering antenatal care determinants from 2018 to 2023.

	2018–2023				
	N	%	PR	95%CI	
**Model 4 +**					
*Antenatal care service*					
Public	12,198	57.77	1.00		
Private	8911	42.20	1.07	1.03	1.11
**Model 4 +**					
*Helth district*					
Reggio Emilia	9454	44.77	1.00		
Scandiano	3191	15.11	0.79	0.75	0.83
Montecchio	2492	11.80	1.20	1.15	1.25
Guastalla	2626	12.44	0.86	0.81	0.90
Correggio	2287	10.83	0.95	0.91	1.00
Castelnuovo ne’ Monti	1065	5.04	0.74	0.68	0.81
**Model 4+**					
*Timing of the first antenatal check*					
Before 12 weeks	19,401	91.88	1.00		
At or after 12 weeks	1711	8.10	0.74	0.68	0.80
Total	21,115				
**Among physiological, model 4+**					
*Professionals*					
Midwife	6248	42.7	1.00		
Obstetrician	8395	57.3	1.00	0.97	1.04
**Among physiological, model 4+**					
*Number of antenatal checks*					
<4	168	1.1	1.00		
4–7	8397	57.3	1.67	1.26	2.22
≥8	6078	41.5	1.69	1.27	2.24
Total	14,643				

PR: prevalence rate ratio. No differences were observed among physiological pregnancies between those followed by a midwife and those followed by an obstetrician.

## Data Availability

Fully anonymized data can be used only for aims linked to assistance, health planning, and quality assurance. Only aggregated data for the full covariant patterns can be made available.
